# SMS-based smartphone application for disease surveillance has doubled completeness and timeliness in a limited-resource setting – evaluation of a 15-week pilot program in Central African Republic (CAR)

**DOI:** 10.1186/s13031-018-0177-6

**Published:** 2018-10-24

**Authors:** Ziad El-Khatib, Maya Shah, Samuel N Zallappa, Pierre Nabeth, José Guerra, Casimir T Manengu, Michel Yao, Aline Philibert, Lazare Massina, Claes-Philip Staiger, Raphael Mbailao, Jean-Pierre Kouli, Hippolyte Mboma, Geraldine Duc, Dago Inagbe, Alpha Boubaca Barry, Thierry Dumont, Philippe Cavailler, Michel Quere, Brian Willett, Souheil Reaiche, Hervé de Ribaucourt, Bruce Reeder

**Affiliations:** 1 Médecins Sans Frontières (MSF), Geneva, Switzerland; 2 Department of Public Health Sciences, Karolinska Institutet, Stockholm, Sweden; 3 World Health Programme, Université du Québec en Abitibi-Témiscamingue (UQAT), Quebec, Canada; 4 Ministry of Health, Bangui, Central African Republic; 5 Country Health Emergency Preparedness & IHR (CPI), WHO Health Emergencies Programme (WHE), WHO, Lyon, France; 6 World Health Organization (WHO), Bangui, Central African Republic; 7 Médecins Sans Frontières (MSF), Stockholm, Sweden; 8 Department of Community Health and Epidemiology, University of Saskatchewan, Saskatoon, Canada

**Keywords:** Surveillance, mHealth, Limited resource settings, Innovation and health

## Abstract

**Background:**

It is a challenge in low-resource settings to ensure the availability of complete, timely disease surveillance information. Smartphone applications (apps) have the potential to enhance surveillance data transmission.

**Methods:**

The Central African Republic (CAR) Ministry of Health and Médecins Sans Frontières (MSF) conducted a 15-week pilot project to test a disease surveillance app, Argus, for 20 conditions in 21 health centers in Mambéré Kadéi district (MK 2016). Results were compared to the usual paper-based surveillance in MK the year prior (MK 2015) and simultaneously in an adjacent health district, Nana-Mambére (NM 2016). Wilcoxon rank sum and Kaplan-Meier analyses compared report completeness and timeliness; the cost of the app, and users’ perceptions of its usability were assessed.

**Results:**

Two hundred seventy-one weekly reports sent by app identified 3403 cases and 63 deaths; 15 alerts identified 28 cases and 4 deaths. Median completeness (IQR) for MK 2016, 81% (81–86%), was significantly higher than in MK 2015 (31% (24–36%)), and NM 2016 (52% (48–57)) (*p* < 0.01). Median timeliness (IQR) for MK 2016, 50% (39–57%) was also higher than in MK 2015, 19% (19–24%), and NM 2016 29% (24–36%) (*p* < 0.01). Kaplan-Meier Survival Analysis showed a significant progressive reduction in the time taken to transmit reports over the 15-week period (*p* < 0.01). Users ranked the app’s usability as greater than 4/5 on all dimensions. The total cost of the 15-week pilot project was US$40,575. It is estimated that to maintain the app in the 21 health facilities of MK will cost approximately US$18,800 in communication fees per year.

**Conclusions:**

The app-based data transmission system more than doubled the completeness and timeliness of disease surveillance reports. This simple, low-cost intervention may permit the early detection of disease outbreaks in similar low-resource settings elsewhere.

## Background

Since 1998, the World Health Organization (WHO) Regional Office for Africa and Member States have adopted the Integrated Disease Surveillance and Response (IDSR) strategy [1]. The Technical Guidelines to support this strategy emphasize the identification of priority diseases using standardized case definitions, reporting mechanisms, epidemiological data analysis and field investigation, outbreak response, communication and feedback [1]. Completeness and timeliness of disease reporting are essential attributes of an effective disease surveillance system [1–3]. Yet, in low-resource settings it is challenging for health facilities to deliver paper-based surveillance reports to national authorities in a timely manner [4, 5]**.** Smartphone applications (known as apps) have been developed to support data transmission between health facilities and district offices in a number of low-income countries, including several in sub-Saharan Africa [2, 6, 7]. Apps have the potential to improve surveillance [8, 9] and to hasten control of potential epidemics. To our knowledge, apps have been used to collect and transmit data on a limited number of diseases [3, 5, 7] or in an emergency context [10, 11] but not on a large portfolio of health conditions in a context of post-conflict insecurity. This report describes the implementation and evaluation of an app surveillance system for the notification of Alerts and transmission of Weekly Reports on 20 conditions from 21 health facilities in the southwestern part of Central African Republic (CAR), in a pilot study entitled “Projet d’Alerte Précoce” (PAP; Early Warning Project, in French).

### Study context and objectives

The district of Mambéré Kadéi (MK) is the second largest health district in CAR with an area of 30,203 km^2^ and estimated population of 460,000. For the purpose of this pilot study, the Ministry of Health (MOH) identified 21 ‘sentinel’ health facilities (3 hospitals, 18 health centers) as these were the principal facilities still functioning within the district and represented all seven sub-districts. Eleven of the health facilities were located in the sub-district of Berberati, two in each of the sub-districts of Gadzi, Gamboula and Amada Gaza, and one in each the sub-districts of Carnot, Dédé Makouba and Sosso Nakombo. The district health office, which includes the office of the Director of Surveillance, is located in the city of Berbérati. Travel time by car or motorcycle from the health facilities to the district health office ranges from 25 min for the closest facility to 9 h for the most distant. Communities in the district are connected by earth roads but lack public systems of water, sewer and electricity. Cellular network service in the district is provided by three telecommunications operators, while villages located on the border with Cameroon are covered by two Cameroonian operators. Only a basic Global System for Mobile communication (GSM) telecommunication service is available.

Health facilities in CAR are expected to submit disease surveillance reports to the district health office according to the national protocol: diseases requiring immediate notification (Alerts) within 24 h; a summary of all notifiable diseases by 5:00 pm Wednesday each week. Both reports are normally transmitted on standard paper forms, although some staff in the health facilities use their own mobile phones and telephone credit to do so.

**Fig. 1:**
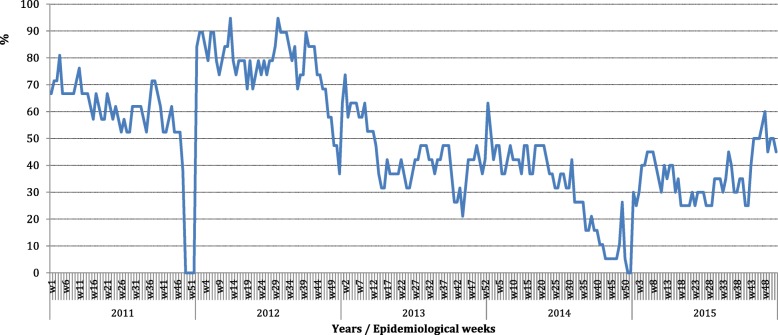
Overview of the Completeness (%)* of Weekly Reports at the District of Mambere-kadei during years 2011 through 2015 [28]. *According to WH0, the accepted cutoff point of completeness (%) is 80% [16]; w = week

In 2013–14 CAR experienced a period of violent conflict between religious and ethnic communities which compromised the health care system, including the disease surveillance system as shown by changes in the level of completeness of disease reporting from 2011 to 2015 (Fig. 1). In some districts of the country, that conflict continues to the present day, while in other districts, such as Mambéré-Kadéi (MK), tensions persist, but only scattered episodes of violence have occurred. During the period of the PAP study (2015–2016), differences in the level of security within the district of MK due to religious violence and non-targeted armed conflict were modest: higher in the more distant sub-districts of Gadzi and Amada Gaza (European Interagency Security Forum (EISF) risk level ‘medium’) and lower in those districts near to the district capital of Bérbérati (EISF risk level ‘low’) [12].

Shortly after the onset of the unrest, Médecins Sans Frontières (MSF) deployed its mission in CAR and in 2015 the MOH and MSF explored how to support the disrupted national disease surveillance system. Due to movement of displaced populations, there was considerable risk of outbreaks of epidemic and vaccine-preventable diseases [13]. To accelerate data transmission and a public health response, MOH and MSF proposed a pilot project using an app reporting system in the district of MK. The main objectives were: 1) to improve the completeness and timeliness of disease surveillance information reaching the district health office, and 2) to evaluate the use of the app to enhance data transmission between the health facilities and the district health office.

## Methods

### The software

The disease surveillance app, Argus, has been developed and made available by the Support to National Surveillance team in the World Health Organization (WHO) Office in Lyon, France [14]. Argus is an integrated solution that comprises a smartphone app and complementary laptop ‘server’ program that permits the reception, validation, analysis and presentation of data in a dashboard format.

The Argus app performs three operations: i) *Alert*, to provide an immediate notification of cases that may signal a potential outbreak; ii) *Report*, to transmit Weekly and Monthly surveillance reports, and iii) *Archive*, to review the status of previously submitted reports. Although the app functions optimally with a 3G cellular telephone network or greater, it requires only a GSM network to transmit basic disease data using a series of Short Message Service (SMS). For an Alert, the numbers of cases, hospitalized patients and deaths are submitted in an open text box format. In the Weekly Reports, the number of cases and deaths for each notifiable disease are submitted as a single SMS; hence a complete report requires a total of 20 SMS messages. Data transmission of the SMS messages occurs through a Universal Serial Bus (USB) modem connected to the laptop computer server maintained by the Director of Surveillance of the health district. The modem contains a Subscriber Identity Module (SIM) card that receives and transfers SMSs into a MySQL database, using Frontline SMS software [15]. After having submitting data to the server, users receive an acknowledgment on their smartphones for each disease notification successfully transmitted. In case of failure in delivery to the server, the app displays an error message on their smartphone screen. In this situation users are required to re-submit the messages.

**Fig. 2:**
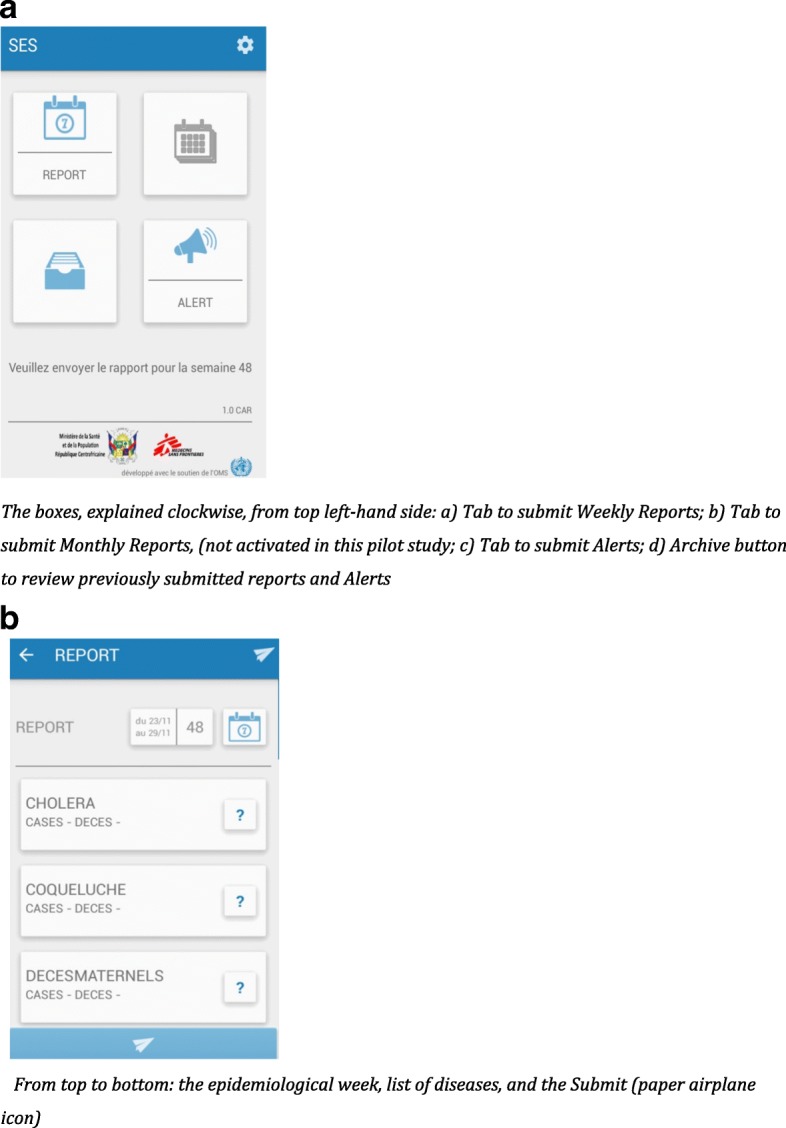
**a** Screenshot of the Main Page of the Argus android phone app*. The boxes, explained clockwise, from top left-hand side: a) Tab to submit Weekly Reports; b) Tab to submit Monthly Reports, (not activated in this pilot study; c) Tab to submit Alerts; d) Archive button to review previously submitted reports and Alerts.* **b** Screenshot of the Report function of the Argus android phone app *From top to bottom: the epidemiological week, list of diseases, and the Submit (paper airplane icon)*

**Fig. 3:**
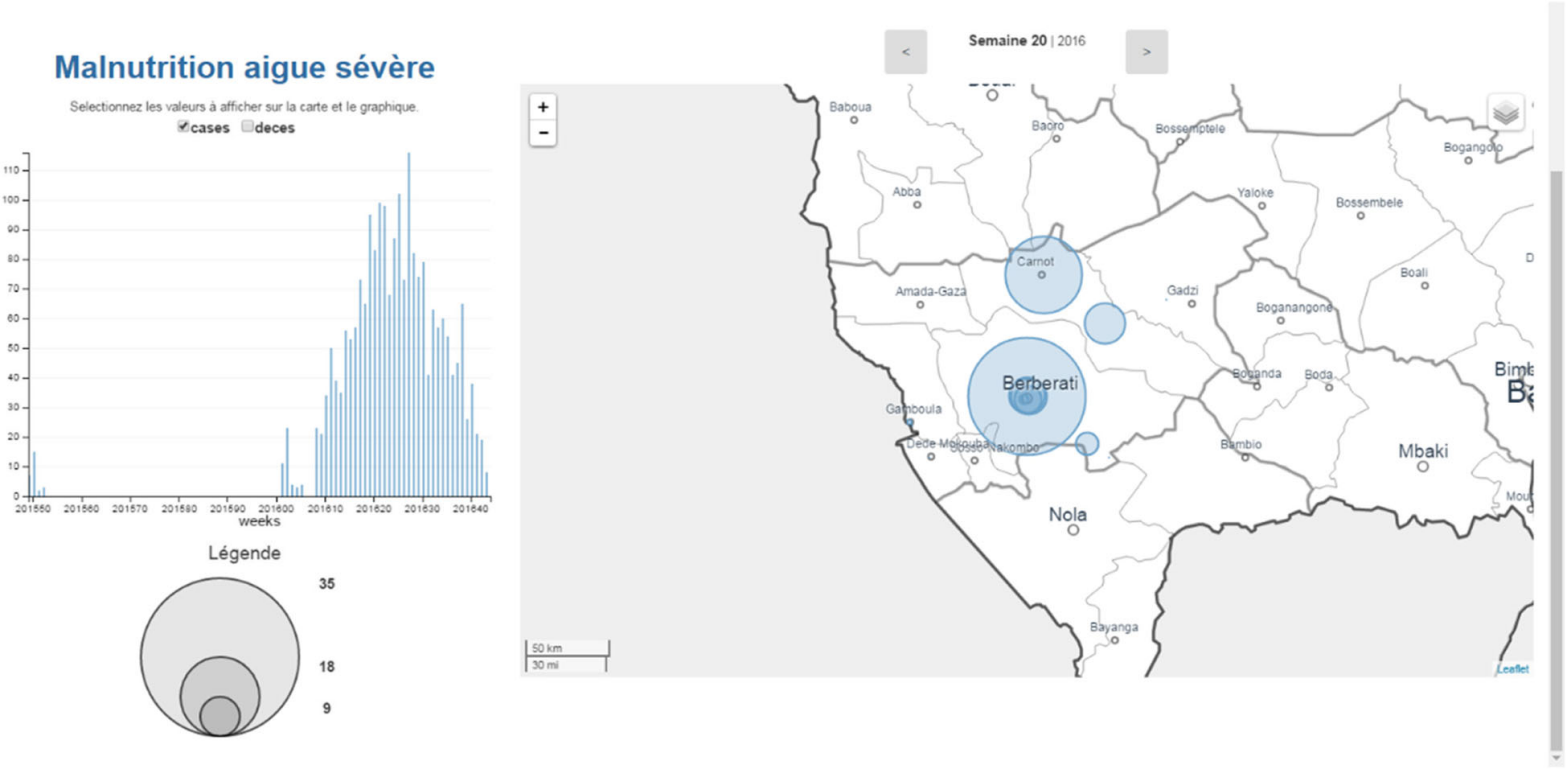
Screenshot of GIS map function of Argus server dashboard showing the district of Mambéré-Kadéi, geographical locations of the health facilities, sub-districts, and cases of acute severe malnutrition during the pilot period

The main page of the app (Fig. 2) displays four tabs: to activate the Alert, Weekly Report, Monthly Report, and Archive functions; the Monthly Report function was not utilized in this pilot. The electronic format of the Alert and Report functions was developed to mirror the existing paper-based disease surveillance report forms in French and designed with a graphical user interface to facilitate data entry, reduce typing, and ensure zero-reporting (Fig. 2b) [16]. Once data has been entered in all 40 required fields (case and death numbers for 20 conditions), users have simply to touch the Submit Report tab (paper airplane icon), and the notifications are sent in the form of 20 individual SMS, in a predetermined standardized format using a comma separator to the server which visually displays the data on a dashboard (Fig. 3). Only reports with data entered in all fields (including the reporting of zeros) are able to be sent by the app. All Alerts and Weekly Reports were reviewed by the Director of Surveillance as soon after receipt as possible. Weekly reports were considered: i) *Complete* if case and death numbers for all 20 conditions were received in the server; ii) *Incomplete* when some, but not all, of the information on the 20 conditions were received by the server; iii) *Rejected* if the Director of Surveillance identified an error in the reports after reviewing them and checking the details of suspected cases with the respective health center by means of an open text SMS or telephone call; or iv) *Validated* when the content of completed reports was deemed to be correct by the Director of Surveillance. With respect to Alerts, the Director of Surveillance investigated the reported cases by telephone with the reporting health facility, however validation of Alerts is not a step included in the app.

### Materials and equipment

The smartphones were Android-based, loaded with the Argus app [17]. Technical support was provided by WHO, Lyon [14, 18], the Geographic Information System (GIS) Unit of MSF and an IT consulting firm based in Geneva [19]. This support comprised programming the app to specify CAR health facilities, telephone and SIM card numbers, diseases under surveillance, and the resolution of periodic technical problems. One full-time local staff member was hired and trained by MSF to provide IT support and to train the Director of Surveillance of the health district to manage the server.

A single smartphone was distributed to each surveillance ‘focal point’ in each participating health facility and a laptop server, together with a backup laptop server, was located in the district health office in Berbérati. Solar energy panels were installed in all health facilities and the district health office to ensure the permanence of power for the mobile telephones and the central server. A contract for a ‘fleet’ of telephone numbers was made with each of the three cellular network providers of service in the district to permit free voice communication between focal points and the Director of Surveillance. The cost of submission of the reports by SMS was covered by prepaid credit with each of the three providers. Two health centers used prepaid Cameroon cellular networks to submit their reports. Finally, in three health centers without mobile telephone network coverage, the cost of motorcycle taxi transport was covered to permit the focal points of these centers to travel weekly to a neighboring village to access the mobile phone network and send their Weekly Report, or to do so at any time to send an Alert.

### Training participants

An initial four-day training workshop on the principles and practice of surveillance was conducted for the surveillance focal points (October 2015). The app was then tested for functionality, using fictitious data, in six health centers in the city of Berbérati (December 2016). Prior to launching the pilot study, the 21 focal points participated in a two-day practical training workshop on electronic data transmission using the app, then received a two-day booster training at week four of the pilot (epidemiological week 14). The booster-training workshop permitted troubleshooting of technical problems and included an agreement by focal points to begin transmission of Weekly Reports early (48 h before the submission deadline). The Director of Surveillance of the health district received training and supervision on the use of Argus on the laptop server for 3 hours weekly during a period of 3 months (March – May 2016). The 15-week pilot study was implemented from 6 March to 11 June, 2016 (epidemiological weeks 9–23).

### Data analysis and study outcomes

We conducted a set of analyses for completeness and timeliness of reporting, duration of transmission, and usability of the app (see definitions in Box) [1]. First, we compared these parameters for MK during the pilot period (epidemiological weeks 9–23, 2016), here termed ‘MK 2016’, with the data for the same district in the same time period the year prior when standard paper-based reporting had been used (epidemiological weeks 9–23, 2015), termed ‘MK 2015’. Second, we compared the data from MK 2016 to that from the district of Nana-Mambéré (NM) during the same time period (epidemiological weeks 9–23, 2016), termed ‘NM 2016’. Wilcoxon rank sum test was used to compare data completeness and timeliness for MK 2016 with MK 2015 and NM 2016. As the surveillance focal points were absent from their health facilities for the booster-training workshop in Berberati during epidemiological week 14, they did not submit their Weekly Reports until returning the following week. Therefore completeness, but not timeliness of reports, was calculated for week 14. For analysis, the 15-week period was divided into five three-week periods and the distribution of transmission times for these periods compared using a Kaplan-Meier survival analysis (Stata/SE Version 12.0 stcox Efron procedure) [20].

The cost of the project was calculated as the total value of expenses [21], including equipment, telephone fleet services, SMS message charges, human resources and Information Technology (IT) consultation services.

**The comparison district, NM,** is a district adjacent to MK, with an area nearly equivalent to MK (26,600 km^2^ versus 30,203 km^2^) and half the population of MK (*N* = 233,666 persons). Both districts share a comparable epidemiological profile and use similar surveillance reporting procedures, however the app was only piloted in MK.

## Results

A total of 3403 cases and 63 deaths were notified in the Weekly Reports during the 15-week period (Table 1). The three most frequent conditions reported were: Severe Acute Malnutrition (30%), Moderate Acute Malnutrition (25%) and Severe Acute Respiratory Infection (17%). The three most frequent causes of death were: Maternal Mortality (33%), Severe Acute Malnutrition (21%) and Severe Acute Respiratory Infection (19%). A total of 26 cases and 4 deaths were notified as Alerts.

**Table 1:** Diseases under surveillance, cases and deaths reported in weekly reports and alerts during the 15-week pilot period, Mambéré Kadéi district 2016

Diseases (alphabetical order)	Weekly Reports				Alerts			
	Cases		Deaths		Cases		Deaths	
	N	%	N	%	N	%	N	%
Acute Flaccid Paralysis	5	0.1%	0	0	2	7^.^7%	0	0
Adverse Events Following Immunization	50	1^.^5%	0	0	1	3^.^6%	0	0
Bacillary Dysentery	66	1^.^9%	0	0	–	–	–	–
Cholera	0	0	0	0	–	–	–	–
Chronic Malnutrition	29	0^.^9%	0	0	–	–	–	–
Diphtheria	1	< 0^.^1%	0	0	–	–	–	–
Dracunculiasis	0	0	0	0	–	–	–	–
Hemorrhagic Fever	0	0	0	0	–	–	–	–
Influenza	215	6^.^3%	1	1^.^6%	–	–	–	–
Malnutrition	–	–	–	–	8	31%	0	0
Maternal Mortality	–	–	21	33.3%	–	–	2	50%
Maternal/Neonatal Tetanus (MNT)	21	0^.^6%	9	14.3%	6	23%	2	50%
Measles	25	0^.^7%	0	0	2	7^.^7%	0	0
Meningococcal Meningitis	78	2^.^3%	5	7.9%	4	15^.^4%	0	0
Moderate Acute Malnutrition	855	25^.^1%	0	0	–	–	–	–
Rabies	12	0^.^4%	1	1^.^6%	3	11^.^5%	0	0
Severe Acute Malnutrition	1018	29^.^9%	13	20^.^6%	–	–	–	–
Severe Acute Respiratory Infection	567	16^.^7%	12	19%	–	–	–	–
Severe Acute Respiratory Syndrome (SARS)	–	–	–	–	–	–	–	–
Typhoid Fever	448	13^.^2%	1	1.6%	–	–	–	–
Whooping Cough	10	0^.^3%	0	0	–	–	–	–
Yellow Fever	3	0^.^1%	0	0	–	–	–	–
TOTAL	3403	100%	63	100%	26	100%	4	100%

*A total of 271 Weekly Reports and 15 Alerts were received during the 15-week pilot period.The sign’ –‘indicates that no data was reported for this condition, whereas the value ‘0′ indicates there were no reported cases of this condition, in compliance with the zero reporting policy of WHO

Over the 15-week period, a total of 530 Weekly Reports and 15 Alerts (together comprising 8378 SMS messages) were received by the server. Of these 530 weekly reports, 271reports (51%) (comprising 5420 SMS) were complete and validated; 71 (13%) were incomplete, and 188 (36%) were rejected. Incomplete and rejected reports were corrected and re-sent by the focal points, sometimes multiple times, thereby increasing the time to report completion; reports would be considered complete and validated only after review and approval by the Director of Surveillance.

**Fig. 4:**
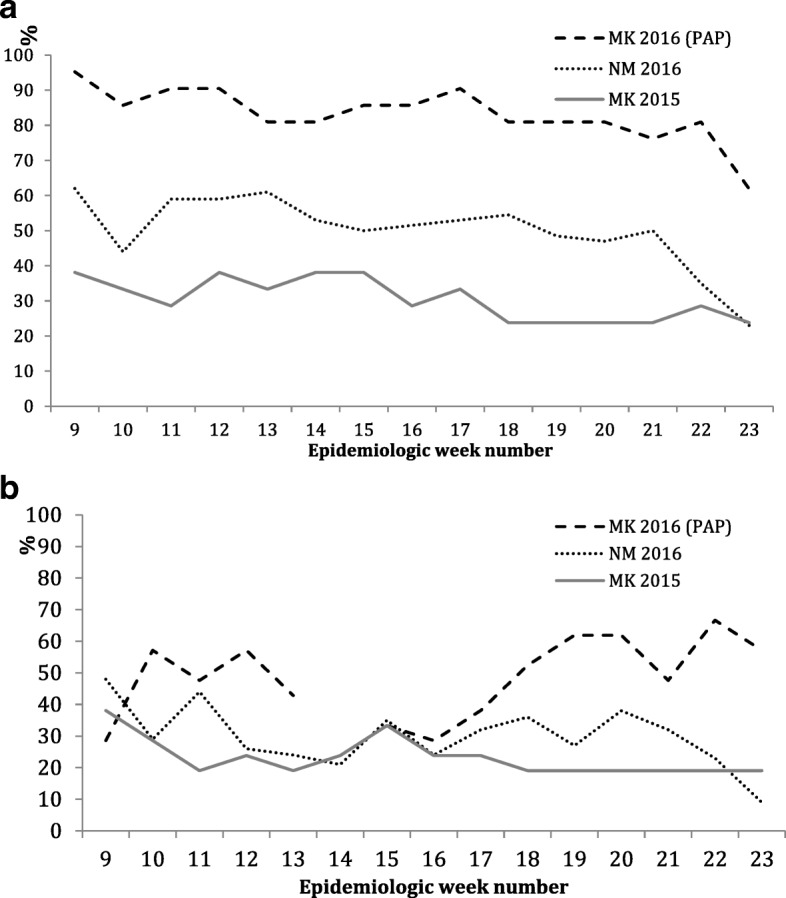
**a** Completeness (%) of Weekly Reports in Mambéré-Kadéi 2016 (PAP), versus paper surveillance in Mambéré-Kadéï 2015 and in the comparison district, Nana-Mambéré 2016**. b** Timeliness (%) of Weekly Reports in Mambéré-Kadéï 2016 (PAP) versus paper-based surveillance in Mambéré-Kadéi 2015 and in the comparison district Nana-Mambéré 2016**.** * p value for both figures was < 0.01 and it was calculated using the Wilcoxon sum test to compare the completeness of Mambéré-Kadéi 2016 (PAP) with the paper surveillance in the districts of Mambéré-Kadéï 2015 and Nana-Mambére 2016

**Fig. 5:**
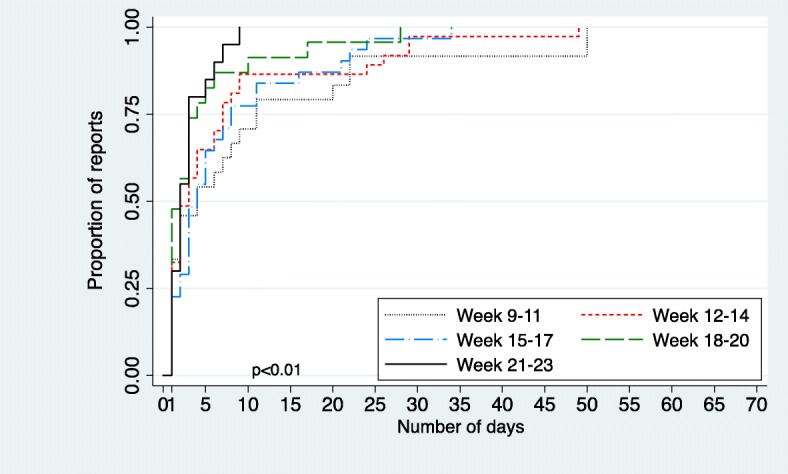
Kaplan-Meier Survival Analysis for the duration of transmission for complete Weekly Reports by 3 week period

Overall, the median completeness of Weekly Reports was significantly higher in MK 2016 (81% (IQR 81–86%)) than in MK 2015 (29% (IQR 24–36%)) and NM 2016 (52% (IQR 48–57%)) (*p* < 0.01; Fig. 4a). Similarly, the median timeliness of complete reports was significantly higher in MK 2016 (50% (IQR 39–57%)), than in MK 2015 (19% (IQR 19–24%)) and NM 2016 (29% (IQR 24–36%)) (*p* < 0.001; Fig. 4b). The median duration of transmission of complete reports was 12 h (IQR 1–63 h). If one were to consider timeliness as the receipt of the *first* SMS transmission of a Weekly Report, the timeliness in MK 2016 would be 74% (IQR 60–80%). Figure 5 shows the Kaplan-Meier survival analysis for time to transmit a complete Weekly Report grouped by 3-week time periods during the pilot. There was a significant progressive reduction in transmission time over the 15-week period (*p* < 0.01).

Consistently each week, health centers across the district initiated electronic transmission of the Weekly Reports as required by the study protocol (96% of occasions); notably, sub-districts showed no significant difference in this regard (Table 2). Yet, completed transmission and report timeliness was significantly lower in a number of sub-districts in both centrally-located low-risk security zones, and in more distant medium-risk security zones. The mean scoring of the usability of the app reported by the 21 focal points was 4^.^5–5/5 for all 27 questions on the three dimensions studied: ease of use, usefulness and efficiency (data not shown). The total cost for the 15-week pilot was US$40,575. This was composed of i) US$10,950 in capital costs: 30 smartphones (US$2250), 30 solar chargers (US$2700), and two laptop computers (US$6000)); ii) US$5200 communication fees (US$1800 fleet services, US$3400 SMS charges); iii) US$225 transportation costs in three isolated health facilities iv) US$4100 local personnel, and v) IT consultant services based in Geneva (US$20,100). It is estimated that to maintain the system in the 21 health facilities of MK will cost approximately US$18,800 per year in communication fees; this represents US$17.20 per health facility per week.

**Table 2:** Level of Weekly Report Completeness and Timeliness by Sub-district

Sub-district	Distance (Km) from district capital (Travel time by motorbike, hours)	Level of security risk (EISF)	Completeness		
			Transmission initiated (%)^a^	Transmission complete (%)^b^	Timeliness (%)^c^
Berberati	5 Km (0.1 h)	Low	153/154 (99.3%)	160/165 (97%)	126/154 (81.8%)
Sosso-Nakombo	58 Km (1–2 h)	Low	14/15 (93.3%)	10/15 (66.7%)	5/14 (35.7%)
Dede-Makouba	71 Km (2–5 h)	Low	29/30 (93.3%)	17/30 (56.7%)	12/28 (42.9%)
Gamboula	86 Km (2–5 h)	Low	30/30 (100%)	26/30 (86.7%)	11/28 (39.3%)
Carnot	76 Km (2–5 h)	Low	15/15 (100%)	14/15 (93.3%)	11/14 (78.6%)
Gadzi	206 Km (5–10 h)	Medium	29/30 (96.7%)	22/30 (73.3%)	16/28 (57.1%)
Amada-Gaza	179 Km (5–10 h)	Medium	21/30 (70%)	13/30 (43.3%)	13/28 (46.4%)
*p* value			*p* = 0.72	*p* < 0.01	*p* < 0.01

^a^The proportion of transmission initiated = Total reports partially or fully received × 100 / total N reports expected from sub-districts)

^b^The proportion of transmission complete = Total reports fully received × 100 / total N reports expected from sub-districts)

^c^As the surveillance focal points were absent from their health facilities for the booster-training workshop in Berberati during epidemiological week 14, they did not submit their Weekly Reports until returning the following week. Therefore completeness, but not timeliness of reports, was calculated for week 14

## Discussion

The objective of the pilot project was to enhance the quality of an integrated strategy for disease surveillance [9] through improved reporting using smartphones. In comparison with paper-based surveillance in both the pilot district the year prior and in a nearby comparison district during the same time period, median completeness and timeliness of reporting from the health facilities more than doubled to 81% and 50%, respectively. Timeliness, itself, progressively improved over the study period.

Mobile phone-based surveillance systems have been pilot tested in several contexts in the past decade. General syndromic surveillance projects in Papua New Guinea [22], Cambodia [2], and Madagascar [23] have produced moderate improvements in the completeness and timeliness of reporting, e.g. an increase in completeness from 40% prior to 70% during the pilot in Papua New Guinea. Targeted surveillance programs for malaria [24], rabies [3], and influenza in Uganda, Tanzania, and Kenya [7], respectively, produced considerable improvements in report timeliness, eg. a reduction in the median reporting delay for influenza in Kenya from 21 to 7 days. Mobile technology appears also to have enhanced surveillance in emergency settings, such as following the earthquakes in Sichuan, China [10] and Haiti [11]. However, larger, comprehensive mobile phone-based surveillance systems have not been assessed in post-conflict settings with ongoing insecurity. This project assesses the impact, feasibility, and cost of using mobile technology for the surveillance of a broad range of notifiable conditions using a simple SMS-based application in a post-conflict setting. Our study shows that, within weeks, the completeness and timeliness of weekly surveillance reports can be considerably improved, and the improvement maintained, despite the remoteness of the healthcare centers and primitive road conditions. Although sub-districts were highly compliant in submitting weekly reports on their mobile devices, the proportion whose transmissions were successfully completed differed significantly (Table 2). Of the two distant sub-districts with the medium-level security risk (Gadzi and Amada-Gaza), one managed a high level of completeness (Gadzi 73.3%) relative to the other (Amada-Gaza 43%). Conversely, two sub-districts in areas of low security risk near the capital (Sosso-Nakombo, Dede-Makouba) managed only 66.7% and 56.7% levels of completeness, respectively. The consistently high level of submission of weekly reports from all sub-districts suggests that remoteness and level of insecurity did not constrain the use of the technology in the health centers. Rather, we suspect that the differences seen between sub-districts in report completeness and timeliness may relate more to the quality of the communications network, the aptitude of the participating staff and, in several settings, the need for motorcycle taxi transport to a nearby community to access a telecommunications network.

The electronic reporting system in the present project was rated as highly usable by participating staff. This may reflect a readiness of the staff to adopt new technology for digital surveillance, however we do not have baseline information with which to measure changes in perceptions or technical skills. The cost of the pilot was higher than mobile disease surveillance interventions reported in other contexts [6]. This is due to the necessary use of smartphones, the provision of these devices to all health facilities, the purchase of ‘fleet’ services from multiple cellular network providers, the costs of SMS transmission, the adherence to the ‘zero reporting’ protocol on a relatively large number of diseases (*N* = 20), and the need for considerable IT consultant services in the developmental stage. The projection of local operating costs beyond the pilot stage, US$17.20 per health facility per week, includes communication fees, but does not estimate the cost of ongoing technical support and staff training. Although still high, this cost is more comparable to that reported elsewhere [2, 3, 7, 22–24].

Several challenges were encountered in this pilot study that require consideration. Smartphones were used in this project to permit use of the app with a user-friendly interface and its associated convenient server dashboard summary of the surveillance data. However, smartphones are not yet commonly used in most sub-Saharan African countries [25]. This necessitated the provision of smartphones to each participating health facility, several days of training prior to the pilot and a booster training at 4 weeks to ensure correct use of the app by healthcare personnel.

The smartphones enabled not only electronic data transmission from health facilities to the district health office via SMS, but since they were configured as a ‘fleet’, telephone contact between the sites was possible to correct errors, provide feedback, and further delineate the epidemiological features of reported cases as needed. The negotiation of ‘fleet’ services with multiple mobile phone service providers proved cumbersome, however, and the project may have been better served at lower cost by the use of a toll-free number to which reports could be sent and calls directed [24].

All submitted Weekly Reports were reviewed and required validation by the Director of Surveillance before entering the server database. Through telephone or SMS contact with the focal points, the Director notified health facilities of incomplete reports that were thereafter re-sent, and investigated details of reports that were rejected. Since reports could only be transmitted once all fields were complete, reports identified in the pilot as ‘incomplete’ were most often incomplete as a result of technical problems during the transmission process. Reports were ‘rejected’ most often as a result of evident data entry errors; these errors were corrected by the focal points and the report resubmitted.

The time taken to transmit complete Weekly Reports in this project was substantial (median 12 h) and highly variable (IQR 1–63). This was due to several factors: i) the poor quality and unreliable nature of GSM telephone network connectivity in the district; ii) the reception of transmissions by a single SIM card of the health district server; iii) the arrival of the bulk of transmission in the few hours before the reports were due. Variation in the proportion of reports that were timely, complete and validated did not differ significantly according to the distance of the healthcare centre from the district office and the associated degree of insecurity, but rather appeared related more to the quality of the communications network and aptitude of the participating staff. It is clear from the progressive reduction in transmission time over the 15-week period that staff quickly became more adept at sending complete, correct reports, and submitting them adequately in advance of the deadline. Participating staff reported developing a sense of comfort and ownership of the electronic surveillance system [26], and could become the future trainers for the implementation of this surveillance program in other districts, should the Ministry of Health proceed to scale-up this project to the national level.

As only a GSM network was available in most of the district, feedback to health facilities on epidemiological trends and issues in the district, and comparison of their performance with that of their peers, could not efficiently be given by such means of an electronic bulletin. Further experimentation is needed to identify a suitable means to do so.

A number of limitations of this work should be recognized. This pilot project focused only on electronic transmission of data from health facilities to the district office; there was no extension to the national level, although the app provides such functionality. Surveillance data that were aggregated and analyzed at the health district office were transmitted verbally by mobile phone to the national office. Clearly, smooth electronic integration of the district program with the national surveillance and health information systems will be vital in the future. We have assessed the impact of smartphones on the quality of disease reporting in a district disease surveillance program. No attempt was made to conduct a comprehensive evaluation of the technology’s effects on the broader dimensions of the IDSR system. According to the national IDSR protocol, cases and deaths of most of the listed conditions are to be notified immediately as Alerts as well as in a summative manner in the Weekly Reports, however, it is apparent from Table 1 that the Alert function is underused. Clearly more intensive training and supervision is required to ensure an immediate reporting of these conditions so as to enable a timely public health response. Comparison was made of the completeness and timeliness of reporting in the district of MK during the pilot period (MK 2016) with that observed in the district during the same time period the previous year (MK 2015). However, the poorer quality of reporting in 2015 may have reflected not only the limitations of a paper-based system, but also the residual effects of civil unrest on the functioning of health facilities in the district at that time. Only a simple cost analysis is presented; a full cost comparison and cost-benefit analysis merits future study. Usability of the system was measured with a short semi-quantitative questionnaire. The responses of the focal points show little variability and the app were ranked highly on all dimensions. It is possible that the instrument used was not adequately sensitive and/or that participants chose to provide a positively biased evaluation of the app to the investigators.

The project was of a limited 15-week duration in a post-conflict setting with only a modest degree of insecurity. Generalization of these results to a setting of more active conflict and greater insecurity should be done cautiously. Finally, it is recognized that sustainability of this pilot initiative and its scale-up to a national level are challenging [8, 27] and will require long-term political commitment, training and resources.

## Conclusion

This SMS-based electronic data transmission system demonstrated a significant improvement in the completeness and timeliness of disease surveillance reports in a remote setting affected by recent conflict, and appears to perform equally well in low and medium risk security zones. This relatively simple intervention may permit the early detection of disease outbreaks in similar settings elsewhere.
